# Late HIV diagnosis is a major risk factor for intensive care unit admission in HIV-positive patients: a single centre observational cohort study

**DOI:** 10.1186/1471-2334-13-23

**Published:** 2013-01-19

**Authors:** Julia Shrosbree, Lucy J Campbell, Fowzia Ibrahim, Phillip Hopkins, Marcela Vizcaychipi, Stephanie Strachan, Frank A Post

**Affiliations:** 1 King's College Hospital, Bessemer Road, London SE5 9RS, UK; 2 King's College London School of Medicine, Weston Education Centre (2.53), Cutcombe Road, London SE5 9RJ, UK; 3 Chelsea and Westminster Hospital, Fulham Road, London, SW10 9NH, UK

**Keywords:** ICU, Intensive care, HIV, Antiretroviral therapy, cART, Immunodeficiency, Late

## Abstract

**Background:**

HIV positive patients are at risk of infectious and non-infectious complications that may necessitate intensive care unit (ICU) admission. While the characteristics of patients requiring ICU admission have been described previously, these studies did not include information on the denominator population from which these cases arose.

**Methods:**

We conducted an observational cohort study of ICU admissions among 2751 HIV positive patients attending King’s College Hospital, South London, UK. Poisson regression models were used to identify factors associated with ICU admission.

**Results:**

The overall incidence rate of ICU admission was 1.0 [95% CI 0.8, 1.2] per 100 person-years of follow up, and particularly high early (during the first 3 months) following HIV diagnosis (12.4 [8.7, 17.3] per 100 person-years compared to 0.37 [0.27, 0.50] per 100 person-years thereafter; incidence rate ratio 33.5 [23.4, 48.1], p < 0.001). In time-updated analyses, AIDS and current CD4 cell counts of less than 200 cells/mm^3^ were associated with an increased incidence of ICU admission while receipt of combination antiretroviral therapy (cART) was associated with a reduced incidence of ICU admission. Late HIV diagnosis (initial CD4 cell count <350 or AIDS within 3 months of HIV diagnosis) applied to 81% of patients who were first diagnosed HIV positive during the study period and who required ICU admission. Late HIV diagnosis was significantly associated with ICU admission in the first 3 months following HIV diagnosis (adjusted incidence rate ratio 8.72, 95% CI 2.76, 27.5).

**Conclusions:**

Late HIV diagnosis was a major risk factor for early ICU admission in our cohort. Earlier HIV diagnosis allowing cART initiation at CD4 cell counts of 350 cells/mm^3^ is likely to have a significant impact on the need for ICU care.

## Background

The advent of combination antiretroviral therapy (cART) has resulted in a marked reduction in the incidence of AIDS and death [1]. Opportunistic infections have become rare in HIV positive patients who are diagnosed at CD4 cell counts above 350 cells/mm^3^ and who initiate cART as per current guidelines. Unfortunately, many patients are only diagnosed with HIV infection once CD4 cell counts have fallen below this threshold or clinical complications have arisen [2]. An initial presentation with AIDS or a CD4 cell count below 200 cells/mm^3^ is now referred to as “advanced disease”, and an initial presentation with AIDS or a CD4 cell count below 350 cells/mm^3^ as “late HIV diagnosis” [3,4]. Both have significant clinical consequences in terms of excess morbidity and mortality [5,6], increased healthcare costs [7,8], and ongoing HIV transmission [2,9]. Applying these definitions, 24–44% of patients in the developed world first present with advanced disease, and 52–59% of patients are diagnosed late [10,11].

Survival rates of asymptomatic HIV positive patients who initiate cART may approach those of the general population. Intercurrent illnesses including opportunistic infections are treated aggressively, if necessary, in intensive care units (ICU). It is estimated that approximately 4–12% of hospitalised HIV patients require ICU admission [12], and 20–40% of those admitted to ICU may be unaware of their HIV status [13-17]. Immunodeficiency is common in HIV positive patients admitted to ICU, with median CD4 cell counts of 39–195 cells/mm^3^ and HIV-related complications present in 21–81% [13-20]. These studies have also reported a low proportion (28–48%) of patients admitted to ICU to be on cART [13-20] or to have undetectable HIV RNA levels (13%) [13]. However, as none of these studies included details of the HIV population from which the patients who required ICU admission arose, the relative contribution of immunodeficiency and the benefit of cART on the incidence of ICU admission remain to be defined.

We investigated the effects of cART and CD4 cell count on the incidence of ICU admission in a large South London HIV clinic. In addition, we examined the contribution of advanced disease and late presentation at HIV diagnosis to ICU admissions.

## Methods

King’s College Hospital is based in South London, UK, and serves a multiethnic HIV population. The hospital provides acute medical and surgical care and specialist treatment to the local population and serves as a tertiary referral centre for South-East England. HIV positive patients are reviewed and monitored with CD4 cell counts and HIV RNA measurements every 3–6 months, and cART is provided free of charge, in accordance with national guidelines. Demographic and clinical characteristics, results of laboratory tests, and antiretroviral treatments are captured in the HIV clinic database.

All patients aged 16 years or older who attended the HIV service between January 2000 and December 2009 were identified in the HIV clinic database which contains demographic parameters including self-identified ethnicity (black vs. white/other), clinical information including AIDS events and prescribed cART, and laboratory parameters including viral hepatitis serology, CD4 cell counts and HIV RNA levels on all patients. HIV positive patients who were admitted to the ICU during the study period were identified by linkage of the HIV and the ICU databases. Patients’ ICU admission diagnoses were categorised as opportunistic or non-opportunistic infections, malignancy, liver or other diseases by the research team. Recurrent ICU admissions in a single patient were regarded as separate events if they occurred at least 3 months apart. Patients admitted to ICU who did not receive their HIV care at King’s College Hospital were excluded from the analyses. The study was approved by the National Health Service Research Ethics Committee and the Research and Development Department at King’s College Hospital.

### Statistical analysis

Data were analysed using STATA (version 11, Stata Corporation, College Station, Austin, Texas). Demographics, clinical and laboratory parameters were described for patients with and without ICU admission during the study period and compared using Chi-squared, Kruskal-Wallis and Student’s T-tests as appropriate.

Generalized estimation equation (GEE) Poisson models with Huber–White sandwich (robust) estimator were used to estimate the crude and adjusted associations between baseline and time updated (CD4 cell counts, HIV RNA and cART) parameters and the incidence of first ICU admission; repeat ICU admissions were excluded from these analyses [21]. Follow-up time was calculated from cohort entry (first outpatient visit or hospital admission at King’s College Hospital after 1/1/2000) and censored at death or the last clinic visit (up to 31/12/2009), and divided into one-month intervals; each interval was assigned the most recent CD4 cell count until a new measurement became available, HIV RNA (<400, ≥400 copies/ml), and cART exposure status (yes/no). The results are presented as incidence rate ratios (IRR) with 95% confidence intervals (CI). All statistical tests are two-sided; associations with P-value <0.10 in univariate analyses were considered to be statistically significant and taken forward into multivariate models.

In separate models, we examined the effects of late HIV diagnosis and advanced HIV disease on early (within 90 days of cohort entry) and late (any time thereafter) ICU admission in patients first diagnosed with HIV infection during the study period. The 90 day cut-off was chosen a priori and considered to reflect the average time from HIV diagnosis required for cART to be initiated and to confer its benefits [22]. These models incorporated age, gender and late HIV diagnosis or advanced HIV disease as fixed covariates, and the most recent CD4 T cell count (late ICU admissions only) and use of cART as time-updated covariates.

## Results

During the study period, 2751 patients received HIV care and were followed for a median (IQR) of 2.27 [0.47, 5.36] years. At the end of the study period, 129 (4.7%) patients were known to have died, and 960 (34.9%) had either transferred their HIV care or were lost to follow up. Of the 2751 patients, 118 (4.2%) required 122 ICU admissions; 24 were newly diagnosed during their ICU admission while an additional 6 patients were diagnosed during their index hospital admission. The characteristics of patients requiring ICU admission and those who were not admitted to ICU are shown in Table 1. Patients needing ICU admission were older, more often female and diagnosed with AIDS, they more often had a history of IV drug use and lower CD4 cell counts at cohort entry. The prevalence of hepatitis B, but not hepatitis C co-infection, was higher among patients admitted to ICU. Nearly half of all ICU admissions were for management of opportunistic infections, with *Pneumocystis jerovecii* pneumonia (n = 22) and tuberculosis (n = 15) the commonest diagnoses. The median (IQR) apache II score on ICU admission was 23 (17,28), and 86% of patients required invasive organ support (mechanical ventilation, renal replacement therapy, vasopressors/inotropes). The median duration of ICU admission was 3 [3,15] days; 45 patients (38%) died in ICU; 51% of patients were discharged from hospital alive.

**Table 1:** Characteristics of HIV positive patients who attended King’s College Hospital, London, UK between January 2000 and December 2009

**Characteristics at cohort entry**^**1,2**^	**All patients (n = 2751)**	**ICU admission (n = 118)**	**No ICU admission (n = 2633)**	**P value**
Age at diagnosis (mean, SD)	35.2 (9.4)	38.4 (9.7)	35.0 (9.3)	0.002
Female sex	1183 (43)	75 (64)	1108 (42)	<0.0001
Black ethnicity	1710 (62)	76 (65)	1634 (62)	0.57
HIV exposure risk factor				0.001
Heterosexual	1645 (60)	77 (69)	1568 (60)	
Homosexual	739 (27)	22 (20)	717 (27)	
IVDU	132 (5)	11 (10)	121 (5)	
Hepatitis B surface antigen positive	134 (7)	12 (12)	122 (7)	0.04
Hepatitis C antibody positive	196 (9)	7 (7)	189 (9)	0.54
AIDS diagnosis^3^	554 (22)	58 (50)	493(19)	0.0001
CD4 cell count (median, IQR)	302 (134, 472)	70 (21, 207)	312 (148, 482)	<0.001
Late HIV diagnosis	1410 (59)	96 (89)	1314 (58)	<0.0001
Advanced HIV disease	810 (34)	79 (73)	731 (32)	<0.0001
**Characteristics of ICU patients**^**1,4,5**^				
CD4 cell count (median, IQR)		81 (21, 191)		
Receiving cART		47 (39)		
HIV RNA <400 c/mL		20 (21)		
APACHE2 score (median, IQR)		23 (17, 28)		
Opportunistic infection^6^		54 (46)		
Non-opportunistic infection^6^		19 (16)		
Malignancy^6^		13 (11)		
Liver disease^6^		6 (5)		
Neurological disease^6^		5 (4)		
Other^6^		21 (17)		

^1 ^Expressed as N (%) unless otherwise indicated.

^2 ^Within 3 months of cohort entry.

^3 ^See methods for definitions.

^4 ^% are based on number of episodes.

^5 ^At the time of first ICU admission.

^6 ^More than one clinical diagnosis may be recorded.

**Figure 1:**
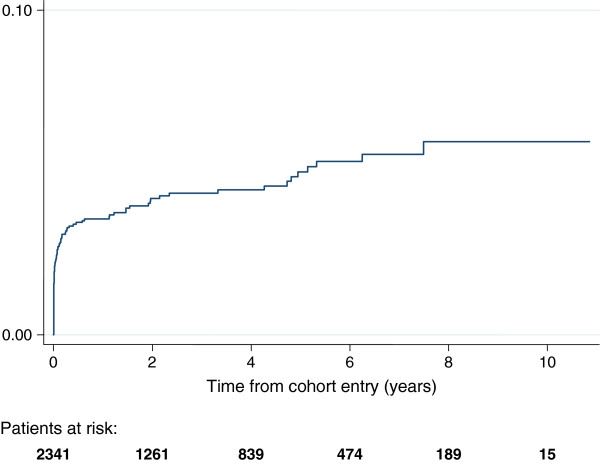
Cumulative incidence (%) of ICU admission among 2341 HIV positive patients attending King’s College Hospital, South London, UK that were diagnosed with HIV infection during the study period.

The overall incidence rate of ICU admission was 1.0 [95% CI 0.8, 1.2] per 100 person-years of follow up. Among 2341 patients first diagnosed with HIV infection during the study period, 57% of ICU admissions occurred within 90 days of HIV diagnosis (Figure 1). The incidence rate of ICU admission in the 3 months following HIV diagnosis was 12.4 [8.7, 17.3] per 100 person-years, and declined to 0.37 [0.27, 0.50] per 100 person-years thereafter (incidence rate ratio 33.5 [23.4, 48.1], p < 0.001). Of the 410 patients diagnosed with HIV prior to January 2000, 15 experienced an ICU admission during the study period, with an incidence rate of 0.46 [0.23, 0.93] per 100 person-years of follow up.

We examined factors associated with ICU admission in multivariate time-updated models incorporating age, gender, HIV risk group, AIDS (CDC-C), use of cART and most recent CD4 cell count. The median time between CD4 cell count measurements was 3.3 [IQR 2.6, 4.4] months and between HIV RNA measurements was 3.2 [IQR 2.4, 4.4] months. AIDS and immunodeficiency (CD4 cell counts <200 cells/mm^3^) were associated with an increased risk of ICU admission, while receipt of cART was associated with a reduced incidence of ICU admission (Table 2). A Sensitivity analysis was performed to identify factors associated with early and late ICU admissions, with very similar findings to the analysis including all ICU admissions (Additional file 1: Supplementary Table).

**Table 2:** Risk factors for ICU admission among 2751 HIV positive patients attending King’s College Hospital, South London, UK

	**Crude IRR**	**P-value**	**Adjusted IRR**	**P-value**
Age (per 10 year increase)	1.39 (1.17, 1.66)	0.001	1.21 (0.95, 1.54)	0.13
Female sex	1.46 (1.22, 2.20)	0.03	1.11 (0.79, 3.48)	0.48
Black ethnicity	0.99 (0.65, 1.52)	0.99		
HIV risk factor				
Heterosexual	1		1	
Homosexual	0.57 (0.33, 0.98)	0.04	0.64 (0.33, 1.25)	0.19
IVDU	1.79 (0.81, 3.95)	0.15	1.01 (0.34, 2.96)	0.99
Hepatitis B surface antigen positive	1.95 (0.99, 3.82)	0.07	1.46 (0.65, 3.28)	0.36
Hepatitis C antibody positive	0.63 (0.23, 1.73)	0.94		
AIDS (CDC-C)	9.58 (5.42, 16.9)	<0.0001	6.94 (3.73, 12.9)	<0.0001
CD4 cell count (cells/mm^3^)				
>350	1		1	
200-350	1.01 (0.48, 2.02)	1.00	0.98 (0.45, 2.15)	0.97
100-200	2.89 (1.39, 5.85)	0.003	2.22 (1.01, 4.88)	0.05
50-100	12.5 (6.35, 24.6)	<0.0001	2.70 (0.92, 7.98)	0.07
<50	21.0 (11.7, 37.8)	<0.0001	6.11 (2.99, 12.5)	<0.0001
Initiated cART	0.17 (0.11, 0.27)	<0.0001	0.11 (0.07, 0.19)	<0.0001
HIV RNA <400 copies/mL	0.73 (0.44, 1.24)	0.31		

CD4 cell count, cART status and HIV RNA level are included as time-updated variables.

cART-combination antiretroviral therapy.

Late HIV diagnosis was present in 81% and advanced HIV disease in 73% of patients who required ICU admission and who were first diagnosed HIV positive during the study period. In multivariable analyses, late HIV diagnosis and advanced HIV disease were strongly associated with ICU admission in the first 3 months following HIV diagnosis (adjusted IRR 8.72 [2.76, 27.5] and 10.90 [4.99, 23.3], respectively), but not with ICU admission thereafter (adjusted IRR 0.95 [0.29, 3.12] and 0.86 [0.33, 2.23]).

## Discussion

Several cohort studies have described the characteristics of HIV positive patients requiring ICU admission [13-20]. To our knowledge, this is the first study to include a denominator population, which allowed us to provide estimates of the contribution of demographic variables and time-updated measures of immune-virological status on the risk of ICU admission. Older age, a history of AIDS and current CD4 cell count were associated with an increased incidence of ICU admission and receipt of cART was found to be highly protective. Late presentation and advanced disease were major risk factors for ICU admission within 3 months of HIV diagnosis. As the majority of ICU admissions occurred during this period, earlier HIV diagnosis and cART initiation at CD4 cell counts of 350 cells/mm^3^ are likely to have a significant impact on the need for ICU care.

The severity of immunodeficiency and low rates of cART use and HIV RNA suppression in our patients are consistent with previous studies of HIV positive patients requiring ICU admission [13-20]. Many complications of HIV infection, including opportunistic infections, non-opportunistic infections, acute renal failure, end-stage kidney disease, liver disease and malignancy are associated with current or past levels of immunodeficiency [21-25]. Thus, it was not surprising to find low CD4 cells count to be associated with ICU admission. The marked reduction in incidence rate of ICU admission after the initial 3 months from HIV diagnosis is consistent with the benefits of cART provision to those with significant immunodeficiency. Our data are also consistent with studies that demonstrated an increased mortality risk in patients who presented with advanced HIV disease, which was most notable in the first 12 months after HIV diagnosis [26,27].

The majority of patients admitted to ICU had a history of late HIV diagnosis. While it is attractive to hypothesise that these admissions could have been avoided by earlier HIV diagnosis, delayed uptake of cART may abrogate the benefits of known HIV status. The UK Collaborative HIV Cohort Study found 37.8% of patients who were diagnosed with CD4 cell counts >350 cells/mm^3^ initiated cART at CD4 cell counts below 200 cells/mm^3^ (late starters) [28]. Although similar outcomes have been observed for “late starters” and “ideal starters” (patients who presented with CD4 cell counts >350 cells/mm^3^ and initiated cART at CD4 cell counts 200–350 cells/mm^3^) after initiating cART [24], the incidence of AIDS and non-AIDS morbidity is significantly increased at CD4 cell counts 200–350 cells/mm^3^[29,30]. Efforts to promote earlier HIV diagnosis to reduce the need for ICU admission may need to be accompanied by efforts to engage those diagnosed with HIV services, to improve uptake of cART among patients with CD4 cell counts <350 cells/mm^3^, and to optimise adherence to cART [31].

This study is limited by its observational nature and retrospective design. In addition, the lack of universal HIV testing in ICU is likely to have resulted in an underestimation of the number of HIV patients requiring ICU admission. The CD4 cell count threshold for cART initiation increased from <200 to <350 cells/mm^3^ during the study period, and adherence and gaps in exposure to cART were not taken into consideration in the time-updated analyses. Finally, although no data were available on potential ICU admissions to other hospitals, King’s College Hospital is the main provider of emergency care in South London and more than 90% of our HIV patients are admitted to King’s College Hospital when they seek emergency care for serious illnesses.

## Conclusion

Immunodeficiency was a major risk factor for ICU admission, and the use of cART was highly beneficial. Earlier HIV diagnosis represents an important opportunity to reduce the need for ICU care and supports current efforts to diagnose those with asymptomatic infection that are unaware of their HIV status.

## Competing interests

In the last 5 years, FAP has received funding for conference attendance, honoraria for membership of advisory boards and research from Abbott, Bristol-Myers Squibb, Gilead Sciences, Janssen-Cilag, MSD, and ViiV Healthcare. All others: no conflict.

## Authors’ contributions

LJC, FI and FAP designed the study. JS identified and reviewed all HIV positive patients admitted to ICU. LJC performed the analyses with input from FI and FAP. JS and FAP wrote the manuscript with input from all authors. The final version of the manuscript was approved by all authors.

## Pre-publication history

The pre-publication history for this paper can be accessed here:

http://www.biomedcentral.com/1471-2334/13/23/prepub

## Supplementary Material

Additional file 1 **Supplementary table. **Risk factors for early and late ICU admission in patients among 2341 HIV positive patients attending King’s College Hospital, South London, UK that were diagnosed with HIV infection during the study period. Click here for file
