# Supplementary material for: Late HIV diagnosis is a major risk factor for intensive care unit admission in HIV-positive patients: a single centre observational cohort study
Source: BMC Infect Dis. 2013 Jan 19;13:23. doi: 10.1186/1471-2334-13-23 (PMC3553027; doi:10.1186/1471-2334-13-23)
Supplement: Additional file 1 — Supplementary table. Risk factors for early and late ICU admission in patients among 2341 HIV positive patients attending King’s College Hospital, South London, UK that were diagnosed with HIV infection during the study period. [file 1471-2334-13-23-S1.doc]

Supplementary Table **Risk factors for early and late ICU admission in patients among 2341 HIV positive patients attending King’s College Hospital, South London, UK that were diagnosed with HIV infection during the study period**

|  | ICU admission within 3 months of HIV diagnosis | | | | | | ICU admission more than 3 months after HIV diagnosis | | | | | |
| --- | --- | --- | --- | --- | --- | --- | --- | --- | --- | --- | --- | --- |
|  | | **Crude IRR** | **P-value** | **Adjusted IRR** | **P-value** |  | | **Crude IRR** | **P-value** | **Adjusted IRR** | **P-value** |  |
| Age (per 10 year increase) | | 1.41 (1.03, 1.93) | 0.03 | 1.16 (0.26, 9.92) | 0.29 |  | | 1.39 (0.99, 1.96) | 0.05 | 1.14 (0.77, 1.69) | 0.51 |  |
| Female sex | | 1.38 (1.15, 2.48) | 0.04 | 0.78 (0.11, 5.96) | 0.62 |  | | 1.83 (0.79, 2.03) | 0.01 | 1.24 (0.89, 6.81) | 0.34 |  |
| Black ethnicity | | 1.32 (0.57, 3.04) | 0.52 |  |  |  | | 1.79 (0.73, 4.38) | 0.21 |  |  |  |
| HIV risk factor | |  |  |  |  |  | |  |  |  |  |  |
| Heterosexual | | 1 |  | 1 |  |  | | 1 |  | 1 |  |  |
| Homosexual | | 0.71 (0.26, 1.92) | 0.50 | 1.38 (0.73, 2.59) | 0.32 |  | | 0.31 (0.09, 1.01) | 0.05 | 0.41 (0.22, 1.21) | 0.31 |  |
| IVDU | | 2.97 (0.97, 9.08) | 0.06 | 1.38 (0.73, 2.60) | 0.59 |  | | 1.49 (0.35, 6.31) | 0.06 | 0.41 (0.09, 1.82) | 0.24 |  |
| Hepatitis B surface antigen positive | | 2.09 (0.62, 7.05) | 0.24 |  |  |  | | 1.99 (0.60, 6.61) | 0.26 |  |  |  |
| Hepatitis C antibody positive | | 0.81 (0.18, 3.58) | 0.78 |  |  |  | | 0.52 (0.70, 3.88) | 0.53 |  |  |  |
| AIDS (CDC-C) | | 8.53 (3.26, 22.3) | <0.0001 | 3.78 (1.46, 9.77) | 0.004 |  | | 8.74 (3.65, 20.9) | <0.0001 | 3.53 (1.49, 8.30) | 0.004 |  |
| CD4 cell count (cells/mm^3^) | |  |  |  |  |  | |  |  |  |  |  |
| >350 | | 1 |  | 1 |  |  | | 1 |  | 1 |  |  |
| 200-350 | | 0.53 (0.06, 5.15) | 0.59 | 1.59 (0.26, 9.92) | 0.50 |  | | 1.42 (0.34, 5.95) | 0.63 | 1.09 (0.24, 4.31) | 0.86 |  |
| 100-200 | | 3.82 (0.85, 17.2) | 0.08 | 2.52 (0.43, 14.9) | 0.31 |  | | 2.44 (1.18, 37.1) | <0.0001 | 2.19 (0.96, 4.99) | <0.0001 |  |
| 50-100 | | 17.3 (4.58, 65.6) | <0.001 | 3.56 (0.54, 23.4) | 0.19 |  | | 5.86 (0.68, 50.4) | 0.11 | 4.83 (0.52, 18.5) | 0.16 |  |
| <50 | | 21.1 (5.93, 75.1) | <0.001 | 11.6 (1.99, 67.1) | 0.006 |  | | 16.4 (7.61, 78.2) | <0.0001 | 6.31 (2.50, 19.7) | <0.0001 |  |
| Initiated cART | | 0.27 (0.12, 0.60) | 0.001 | 0.20 (0.08, 0.49) | <0.0001 |  | | 0.39 (0.18, 0.80) | <0.0001 | 0.24 (0.11, 0.52) | <0.0001 |  |
| HIV RNA <400 copies/mL | | 0.41 (0.09, 1.80) | 0.24 |  |  |  | | 1.20 (0.67, 2.19) | 0.51 |  |  |  |
